# Soluble guanylyl cyclase maturation in cells depends on reduction of the incoming heme by a cysteine in the enzyme’s beta subunit

**DOI:** 10.1016/j.jbc.2026.113541

**Published:** 2026-09-08

**Authors:** Yue Dai, Saurav Misra, Dennis J. Stuehr

**Affiliations:** 1 Department of Inflammation and Immunity, Cleveland Clinic Research, Cleveland, Ohio, USA; 2 Department of Biochemistry and Molecular Biophysics, Kansas State University, Manhattan, Kansas, USA

**Keywords:** cGMP, GAPDH, heat shock protein 90, heme trafficking, electron transfer, cell redox, thiol redox, nitric oxide

## Abstract

Nitric oxide (NO) activates cGMP production by soluble guanylyl cyclase (sGC). To do so, an sGCβ subunit must incorporate heme and form a heterodimer with a partner α subunit. In cells, heme-free (apo) sGCβ obtains heme from glyceraldehyde 3-phosphate dehydrogenase (GAPDH). We recently found that transfer of ferric heme from purified GAPDH to apo-sGCβ was accompanied by heme reduction to ferrous within sGCβ, implicating redox involvement of its Cys residues. Here, we utilized Ser substitutions to probe three conserved, redox active Cys in sGCβ (C78, 122, and 214). Proteins were studied in live HEK293 cells and in purified form. In cells, C78S sGCβ was unique among the variants in being unable to incorporate either added ferric heme or mitochondrially-generated heme, but it did incorporate added ferrous heme-NO and consequently formed an active sGC heterodimer. An internal Cys oxidation event occurred in the wild-type, C122S, and C214S apo-sGCβ proteins during their uptake of ferric heme from GAPDH but did not occur in C78S apo-sGCβ. We conclude: (*i*) Mitochondrial heme reaching apo-sGCβ in cells is ferric under normal culture conditions. (*ii*) C78 in the sGCβ subunit provides a reducing equivalent to the incoming ferric heme to reduce it to ferrous and this enables stable heme incorporation. (*iii*) No other Cys residues in sGCβ or cell thiols can replace the role of C78. Having a protein’s heme incorporation rely on an internal Cys redox event is unusual and suggests how sGC maturation may be linked to the thiol redox status in cells.

Nitric oxide (NO) regulates numerous physiological and cellular functions including vasorelaxation (1), peristalsis (2), immune surveillance (3), reproductive processes (4), bronchodilation (5), and neural communication (6) by binding to its receptor enzyme soluble guanylyl cyclase (sGC) and stimulating its cyclic guanosine monophosphate (cGMP) generation (7, 8). Because NO binds to a ferrous heme cofactor located in the sGCβ subunit of the sGCα/β heterodimer (9, 10), it is important to understand the cellular processes that enable and regulate sGC heme acquisition. To this end, we have exploited sGCβ reporter constructs developed by Hoffmann *et al.* (11) that utilize fluorescein arsenical hairpin binder (FlAsH) technology developed by Tsien and colleagues (12) to measure sGC heme acquisition. These sGCβ reporter constructs have a tetra-Cys–containing sequence (TC) inserted near the sGCβ heme-binding site, thus creating TC-sGCβ (13). When the TC sequence is labeled with the arsenical dye FlAsH (14), the TC-sGCβ protein becomes fluorescent and its emission intensity is inversely proportional to its bound heme content. This has allowed us to track time-resolved changes in heme content of purified TC-sGCβ reporter proteins *in vitro* as well as in TC-sGCβ expressed in live mammalian cells (11, 13, 15, 16). Among other findings, we have observed that newly translated sGCβ subunit is heme-free (apo-sGCβ) and associates with heat shock protein 90 (Hsp90) (13, 17, 18). Hsp90 is a major chaperone that generally acts through conformational mechanisms to enable the folding and conformational maturation of numerous protein clients as well as to promote client binding of small ligands or cofactors (19). Glyceraldehyde-3-phosphate dehydrogenase (GAPDH) transports and delivers the intracellular heme to the apo-sGCβ-Hsp90 complex (15, 20), and Hsp90 then drives an ATPase-dependent heme insertion into apo-sGCβ (13, 17, 21). Hsp90 then dissociates from the heme-replete sGCβ subunit, allowing the sGCβ to associate with an sGCα subunit to form the mature and functional sGCα/β heterodimer (21).

To act as an NO-binding sensor, the sGCβ heme must be in the ferrous redox state. But the extent to which the heme redox state may influence heme incorporation into apo-sGCβ, and how the heme becomes ferrous, is still unclear. In experiments utilizing a defined *in vitro* heme transfer system with purified GAPDH, Hsp90, and apo-sGCβ proteins, we recently observed that ferric heme transfer from GAPDH into apo-sGCβ is accompanied by reduction of the heme to its ferrous state (15). Furthermore, our findings suggested that the reducing equivalent provided to the incoming ferric heme was provided by the apo-sGCβ protein itself. Because this implies possible redox involvement of the protein Cys residues, here we investigated three Cys residues in sGCβ as potential sources of the reducing equivalent. Structures of the sGCα/β heterodimer (9, 10) show two of these Cys are located quite near the heme (C78, C122) while the other is at a greater distance (C214), and none are in position to form disulfide bonds. Our findings identify C78 as the sole source of the reducing equivalent that converts the incoming ferric heme to ferrous during its uptake into apo-sGCβ in both live mammalian cells and in the *in vitro* reconstitution system. This C78-driven redox step is required for apo-sGCβ to incorporate mitochondrially generated heme in living cells, which in turn enables the formation of functional sGCα/β heterodimers. Thus, the C78-mediated redox step explains how the heme in sGC becomes ferrous during its maturation to functional form, and also provides a new potential means whereby the cell’s thiol redox status can regulate this process (22).

## Results

### Cellular heme incorporation into apo-sGCβ requires Cys78 and is regulated by the heme redox state

The human and rat sGCβ subunits each contain 14 Cys residues, including three that are highly conserved in animals (Fig. S1) and that are located either in the heme-binding domain (C78, C122) or in the Per-Arnt-Sim (PAS) domain (C214). Based on Cryo-EM structures of sGCα/β heterodimers (PBD 6JT0, 6JT1, 6JT2) (9) (Fig. 1), the sulfurs in these three Cys are approximately 4, 12, and 37 Å away from the nearest heme edge, respectively, and none are in position to form disulfides. Previous studies implicated C78 and C214 as regulators of heme incorporation into sGC overexpressed in insect cells (23) and showed that C78 and C122 can be redox active within sGC (22, 24). Thus, we used our experimental systems to investigate the influence of C78, C122, and C214 on heme incorporation into apo-sGCβ. We made individual Cys to Ser substitutions at C78, C122, or C214 in our full-length TC-sGCβ (residues 1-619; designated sGCβ) or in our truncated TC-sGCβ (residues 1-359) reporter proteins as previously described (13). We studied the full-length TC-sGCβ variants expressed in the human cell line HEK293 and investigated the shorter recombinant (*Escherichia coli* expressed) TC-sGCβ variants in their purified forms.

**Figure 1:**
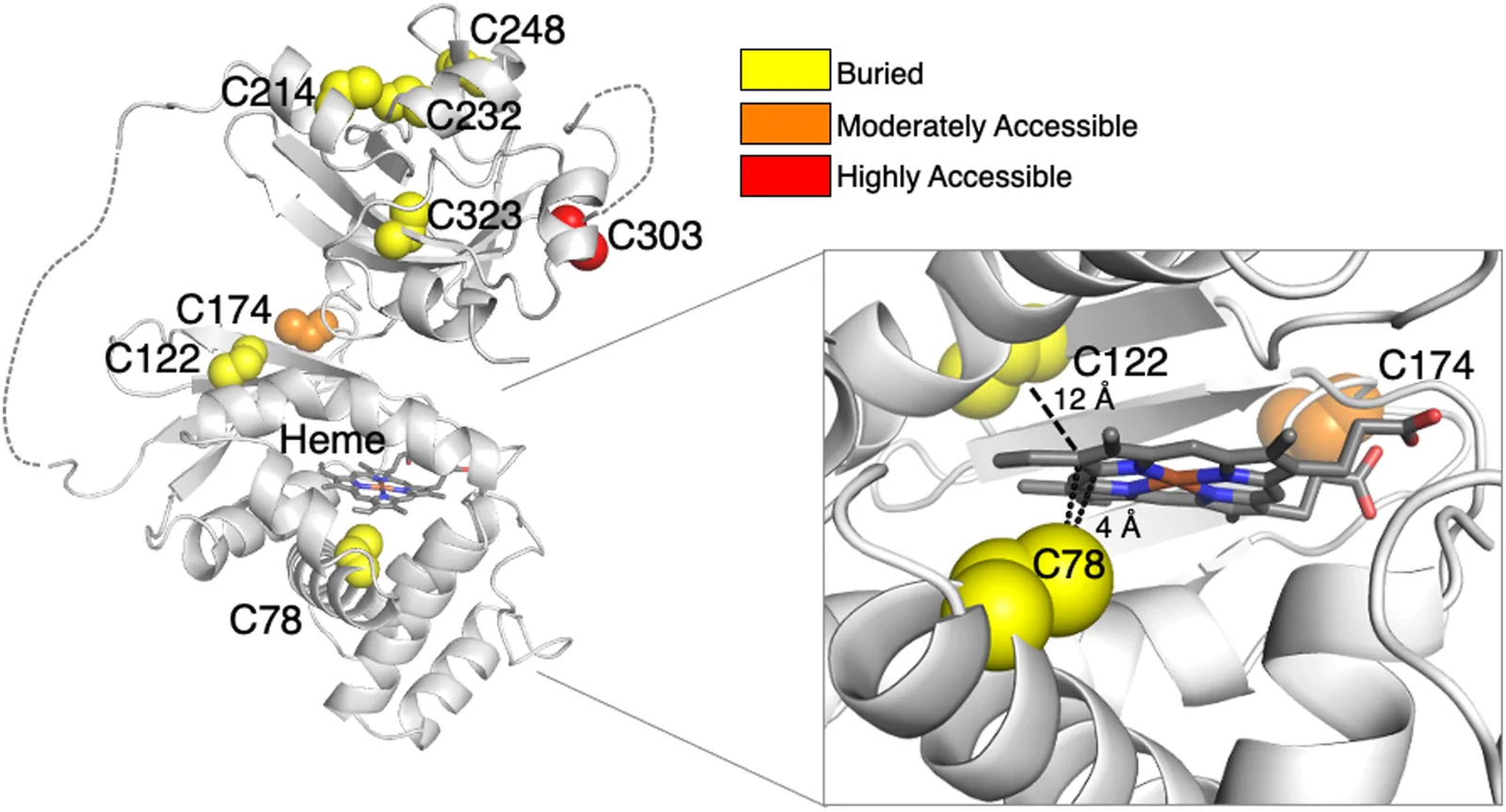
**Locations of Cys in the heme-binding and PAS domains of human sGCβ (from PDB**6JT0**).** The structure shown for sGCβ is as it occurs in the sGCα/β heterodimer. Cys side chains are shown as spheres and color coded based on solvent accessibility as determined using GETAREA. The inset shows a close-up view of the heme and the indicated Cys residues, with the distances from the C78 and C122 sulfur atoms to the heme edge indicated.

Heme proteins expressed in mammalian cells under typical culture conditions accumulate as a mixture of their heme-containing and heme-free forms (25). This also holds for sGCβ and TC-tagged sGCβ, with their apo forms typically predominating (16, 17, 21, 25). Thus, the dynamics of cellular heme incorporation into apo-sGCβ can be studied under normal culture conditions either by stimulating mitochondrial heme biosynthesis by adding the heme precursors δ-aminolevulinic acid and Ferric citrate (δALA/Fe) or by adding exogenous ferric heme to the cultures (13, 20, 21). The heme insertion kinetics can be studied with our established FlAsH-labeled apo-TC-sGCβ probe whose fluorescence is quenched by approximately 60% upon heme incorporation (Fig 2*A*). We expressed wild-type TC-sGCβ or each corresponding C78S, C122S, and C214S variant in HEK293 cells, FlAsH-labeled them, and then measured sGCβ heme incorporations by monitoring cell fluorescence in a 96-well plate reader (Fig. 2*B*). Figure 2*C* shows averaged fluorescence traces recorded after δALA/Fe was added to the HEK293 cultures at time = 0. Cells expressing wild-type, C122S, or C214S TC-sGCβ all showed similar gradual decreases in their FlAsH fluorescence over 30 min, indicating that their TC-apo-sGCβ subpopulations steadily incorporated the heme generated by cell mitochondria in response to δALA/Fe addition. Addition of the sGCβ heme oxidant 1H-[1,2,4]oxadiazolo-[4, 3-a]quinoxalin-1-one (ODQ) at the point of δALA/Fe addition prevented the cell heme incorporations into each of the three sGCβ species (Fig. 2*C*), matching the ODQ inhibitory effect that we had previously observed for ferric heme transfer from GAPDH into purified apo-TC-sGCβ (26). We then tested if the ODQ addition inhibited the cell’s mitochondrial heme production or its availability to GAPDH, and found that it did not, as judged from it not impacting mitochondrial heme loading into FlAsH-labeled TC-GAPDH in response to the δALA/Fe addition to the cells (Fig. S2). In comparison, HEK293 cells expressing the FlAsH-labeled C78S TC-sGCβ variant exhibited no fluorescence decrease upon addition of δALA/Fe either in the absence or presence of ODQ (Fig. 2*D*). Together the results suggest that successful sGCβ heme incorporation in live cells requires that the heme accumulate within the sGCβ subunit in its ferrous form (which is rapidly oxidized by the ODQ), and also show that among the three TC-sGCβ variants we tested, only the C78S variant failed to incorporate mitochondrially-generated heme.

**Figure 2:**
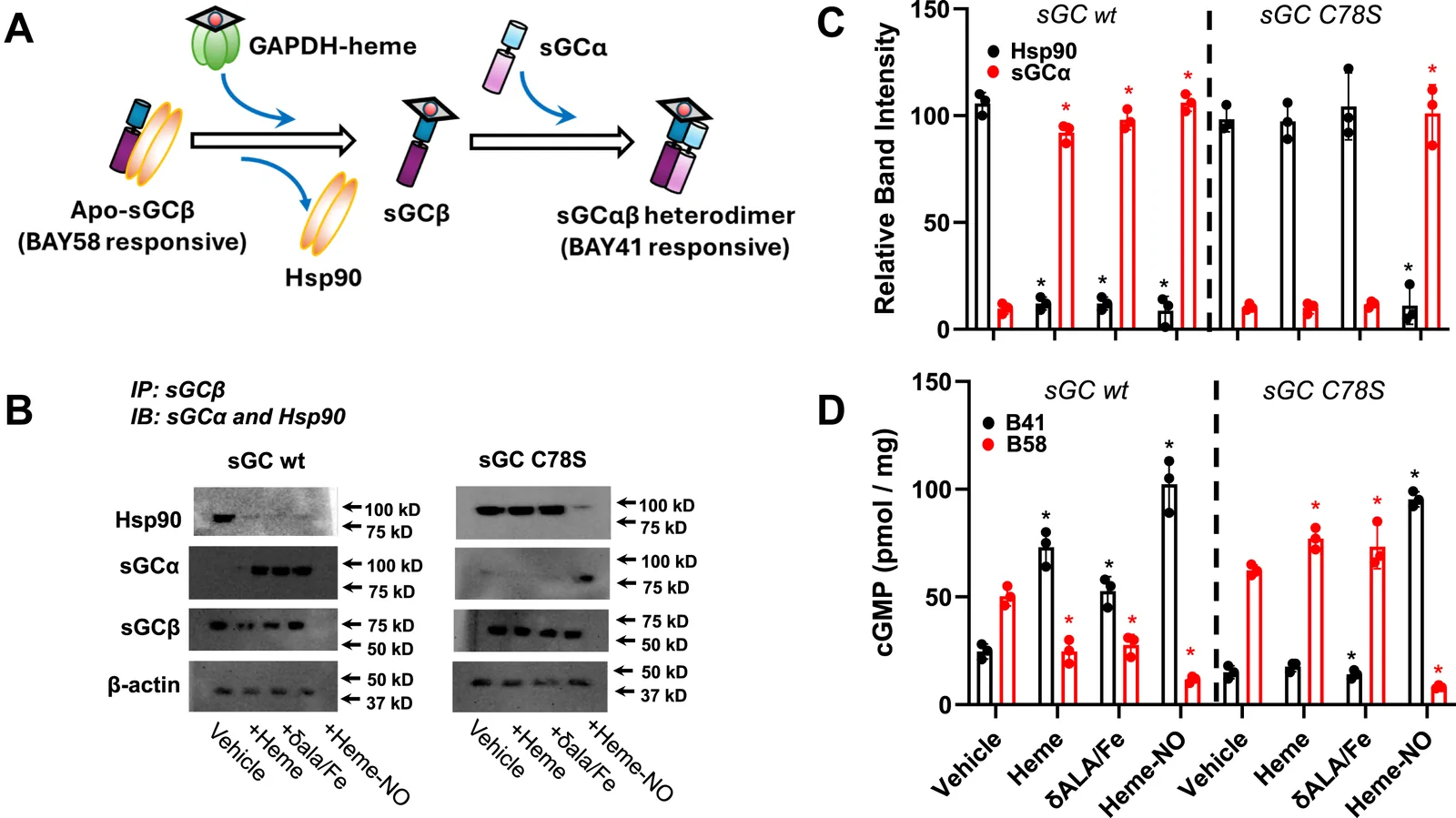
**Kinetics of heme uptake into TC-apo-sGCβ wild type or the Cys to Ser variants in live HEK293 cells following stimulation of cell mitochondrial heme biosynthesis.** Wild-type TC-sGCβ or each Cys to Ser variant was expressed in HEK293 cells and FlAsH labeled. *A*, cartoon indicating the fluorescence quenching that occurs when heme incorporates into a FlAsH-TC-apo-sGCβ protein. *B*, cell fluorescence was monitored with time after adding δ-ALA plus Fe-cit (δALA/Fe) to the cultures. In some cases, the sGCβ heme oxidant ODQ was also added along with δALA/Fe, or the cells received ferric heme or ferrous heme-NO in place of δALA/Fe, as indicated. *C*, *D* and *E*, fluorescence intensities *versus* time recorded for the indicated cell cultures. Cells received δALA/Fe and ODQ as indicated in paels (*C*) and (*D*), and received ferric heme or ferrous heme-NO as indicated in (*D*). RFU, relative fluorescence units. The traces are the mean ± SD of three wells and are representative of three independent experiments.

We next examined if the defective heme uptake by C78S apo-TC-sGCβ in the cells was related to the redox state of the incoming heme. To do this, we utilized a pre-formed ferrous heme-NO compound (15) that we previously showed is redox-stable in aerated culture solutions and can enter cells and be delivered intact into apo-sGCβ without loss of NO or a change in the heme redox state (15, 27, 28). The C78S apo-TC-sGCβ variant did not incorporate ferric heme when it was added to the cells (Fig. 2*E*), but it did incorporate exogenously added ferrous heme-NO compound in a manner equivalent to the wild-type TC-sGCβ (Fig. 2*E*). We observed an identical discrimination pattern when the supernatants made from cells that expressed the FlAsH-labeled versions of wild-type apo-TC-sGCβ or each individual variant were titrated with either ferric heme or the ferrous heme-NO species (Fig. 3). We confirmed that the cell expression levels of wild-type or variant TC-sGCβ proteins were similar in each of the comparative experiments described above (Fig. S3). Thus, C78S apo-TC-sGCβ was specifically defective in incorporating mitochondrially generated heme or incorporating exogenously supplied ferric heme, but it could incorporate a stable ferrous form of heme (heme-NO) as well as did the wild-type apo-TC-sGCβ, indicating its defect in heme incorporation is only related to the redox state of the incoming heme.

**Figure 3:**
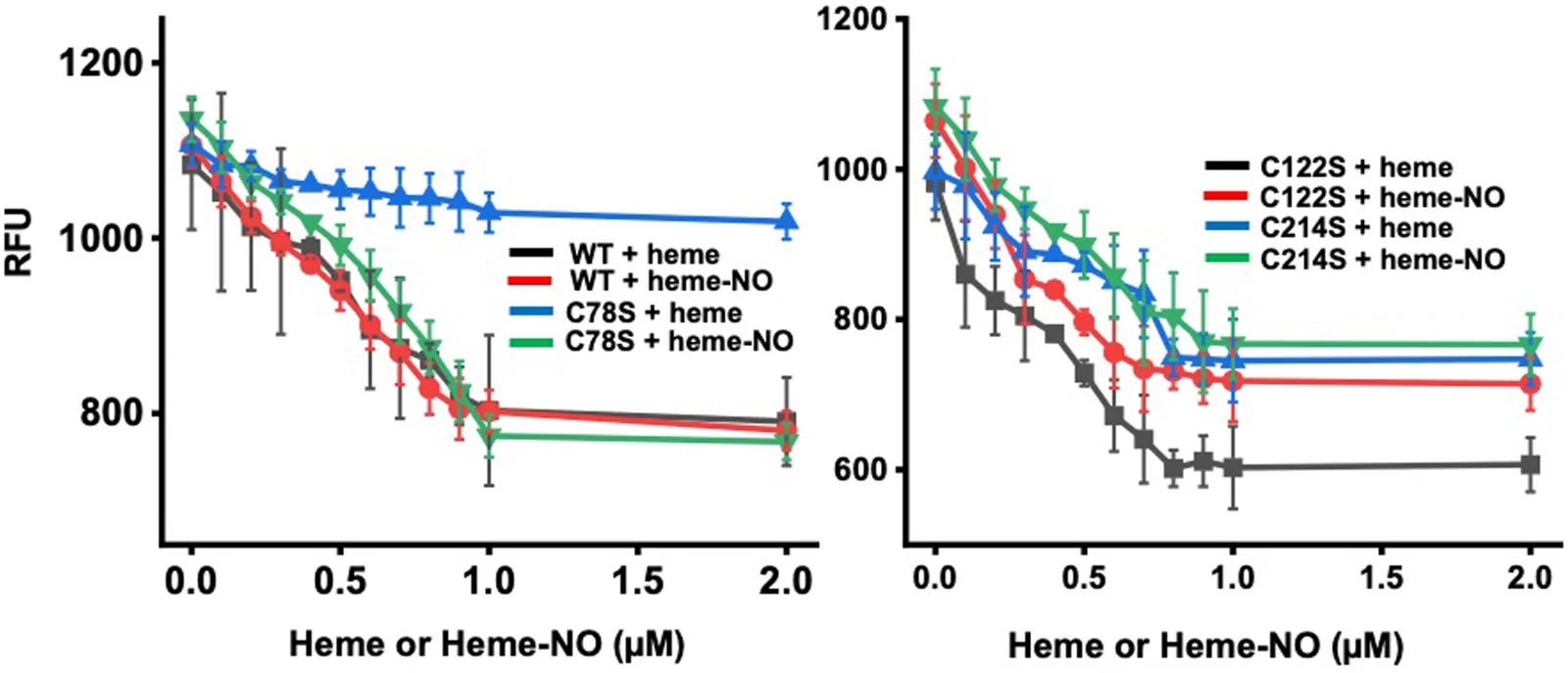
**Titration of cell supernatants containing the indicated FlAsH-labelled apo-TC-sGCβ proteins with ferric heme or ferrous heme-NO.** Cell supernatant solutions made from cells expressing each indicated sGCβ protein received vehicle or the indicated heme species to give the indicated final heme concentrations, and then fluorescence readings were recorded. Heme, ferric heme; heme-NO, ferrous heme NO; RFU, relative fluorescence units; WT, wild type. The traces are the mean ± S.D. of three wells and are representative of three independent experiments.

### Cys78-mediated heme incorporation enables maturation of a functional sGC heterodimer

We next studied how the aberrant heme incorporation into C78S apo-sGCβ would impact two key steps in sGC maturation that normally follow heme incorporation and are required for it to gain biological function (Fig. 4*A*), namely, dissociation of Hsp90 chaperone from sGCβ and its subsequent binding with an sGCα subunit to form a heme-containing sGCα/β heterodimer. Thus, HEK293 cells that were expressing sGCα plus either wild-type or C78S apo-sGCβ were treated for 1 h with vehicle alone or vehicle plus either δALA/Fe, ferric heme, or ferrous heme-NO, followed by cell supernatant preparation. We used immunoprecipitation to investigate how the various treatments would impact the relative levels of Hsp90 and sGCα subunit associations with the wild type and C78S sGCβ subunits. As shown in Figure 4, *B* and *C*, in the cells that received only vehicle the Hsp90 associations with the wild-type or the C78S sGCβ subunits were relatively strong and the sGCα associations were relatively weak. For wild-type sGCβ, its level of Hsp90 association was diminished by 8 to 10-fold in the supernatants prepared from cells that had received either δALA/Fe, heme, or ferrous α heme-NO, and these reductions in the Hsp90 association level coincided with 7 to 11-fold increases in sGCα association. Thus, as expected, all three methods of heme provision stimulated Hsp90 to dissociate from wild-type apo-sGCβ and promoted its association with sGCα to form a sGCα/β heterodimer. In contrast, for the C78S apo-sGCβ, we only observed a loss in its Hsp90 association and gain in its sGCα association in supernatants prepared from cells that had received ferrous heme-NO.

**Figure 4:**
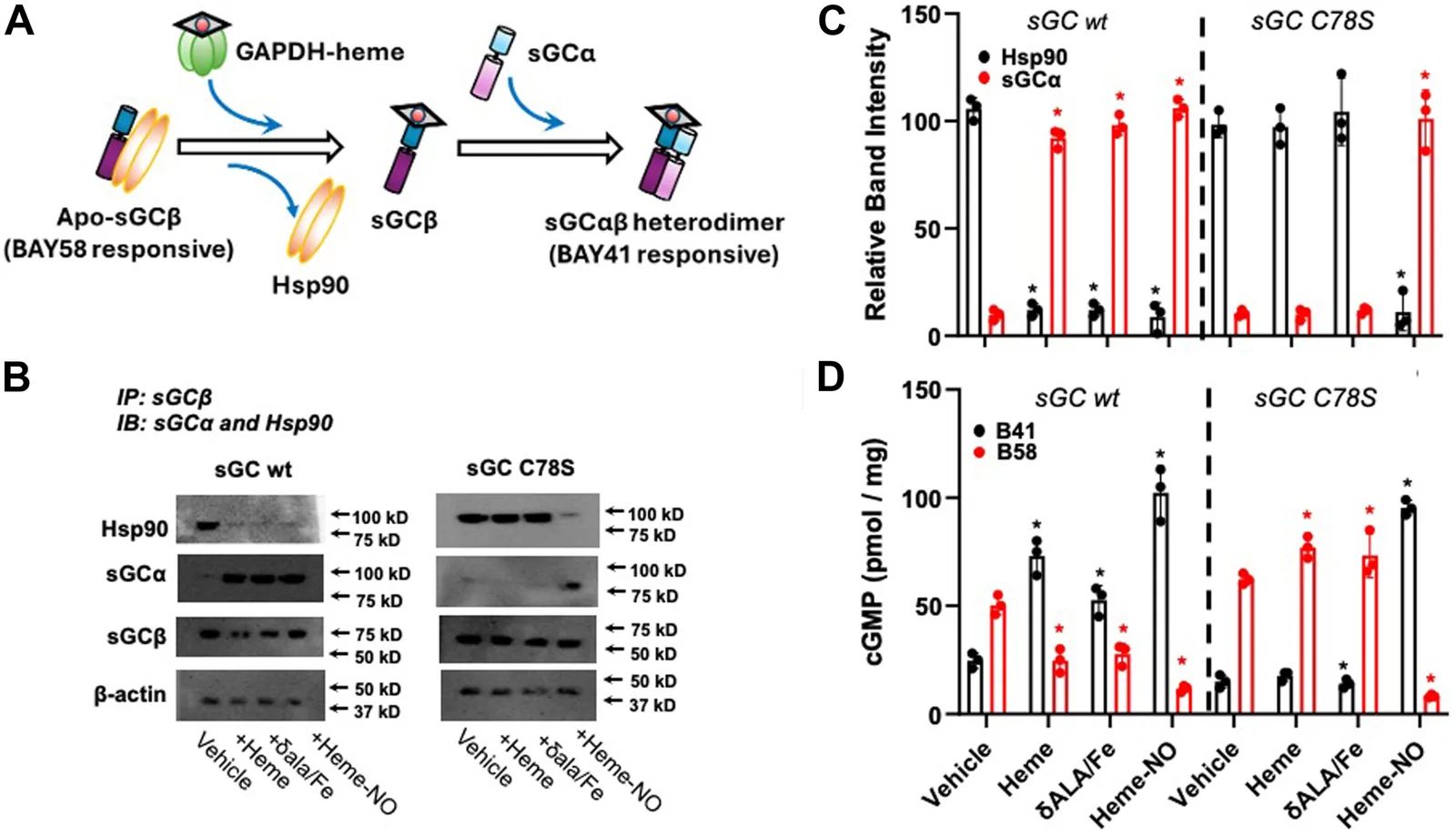
**Capacities of wild-type and C78S apo-sGCβ to form a functional sGC heterodimer in HEK293 cells in response to different heme species.** HEK293 cells were transfected to express sGCα and either wild type or C78S sGCβ and were then given either ferric heme (Heme), δALA/Fe, or ferrous heme-NO (Heme-NO) and were further incubated for 30 min before being lysed and supernatants prepared. *A*, the changes in apo-sGCβ protein partner and BAY58 or BAY41 drug responses that normally occur upon it incorporating heme and becoming a functional heterodimer in mammalian cells. *B*, representative Western blots indicating the relative amounts of Hsp90 and sGCα bound to sGCβ in IP’s prepared from the indicated cell supernatants, and the relative amounts of sGCβ and β-actin present in the supernatants. *C*, densitometric quantification of Hsp90 and sGCα bands in the sGCβ IP’s. *D*, cGMP generation levels by the indicated supernatants in response to either BAY41 or BAY58. Values in Panels *C* and *D* are the mean ± SD of three independent experiments and are compared to vehicle condition values for significance determination. GraphPad Prism 10 was used to evaluate the differences between groups by a one-way ANOVA. Dunnett's test is used as *post hoc* test. ∗*p* < 0.05 was considered statistically significant in all analyses.

Another consequence of maturation to the heme-containing sGC heterodimer is that its cGMP synthesis is able to become enhanced in response to the heme-dependent sGC activator BAY 41-2272 (BAY41), while it becomes depressed in response to the heme-independent activator BAY 58-2667 (Cinaciguat, or BAY58) (14, 18, 19, 29). Thus, upon measuring cGMP generation by the same cell supernatants in response to BAY41 *versus* BAY58, we could confirm the different effects that the various heme provisions had on wild-type and C78S sGCα/β heterodimer formation (Fig. 4*D*). Together, these results show that despite having refractory responses to endogenously generated or added ferric heme, the C78S apo-sGCβ was perfectly capable of forming a functional sGCα/β heterodimer in cells as long as the cells were provided with the ferrous heme-NO species.

### C78 is needed for purified apo-sGCβ to incorporate ferric heme

To characterize the Cys-dependent redox mechanism that is enabling the sGCβ heme incorporation, we generated the Cys variants in our truncated, bacterially expressed and purified sGCβ reporter protein (aa 1-385; TC-sGCβ_1-385_), which contains the heme-binding and PAS domains as well as a short segment of the helical extended domain. We and others have previously used this well-expressed and stable protein construct to effectively elucidate various aspects of sGCβ biochemistry, including its heme uptake from GAPDH and Hsp90 binding (15, 21, 30). We thus utilized the purified FlAsH-labeled TC-sGCβ_1-385_ variants and compared their abilities to take in ferric heme *versus* ferrous heme-NO when they were either titrated in or were provided as pre-formed GAPDH complexes. As shown in Figure 5, unlike wild-type apo-TC-sGCβ_1-385_ or the C122S and C214S variants, the C78S variant did not incorporate ferric heme upon titration but readily incorporated ferrous heme-NO to a similar extent as did wild type. In separate experiments that studied the kinetics of the transfer from pre-formed GAPDH-ferric heme or GAPDH-ferrous heme-NO complexes, we found that the apo-TC-sGCβ_1-385_ C78S variant was also unable to incorporate the ferric heme from GAPDH, but it incorporated ferrous heme-NO from GAPDH as well as did wild type or the other two apo-TC-sGCβ_1-385_ variant proteins (Fig. 6, *A* and *B*). In similar experiments performed under an anaerobic atmosphere, the C78S apo-TC-sGCβ_1-385_ also did not incorporate ferric heme from a GAPDH heme complex while it did incorporate ferrous heme from GAPDH in a manner similar to wild type apo-TC-sGCβ_1-385_ (Fig. 6, *C* and *D*). Thus, the heme incorporation behaviors of the purified truncated apo-sGCβ proteins matched results we obtained for their full-length counterparts in the live HEK293 cells, and confirmed in the defined *in vitro* system that C78 is critical for apo-sGCβ to incorporate ferric heme provided by GAPDH.

**Figure 5:**
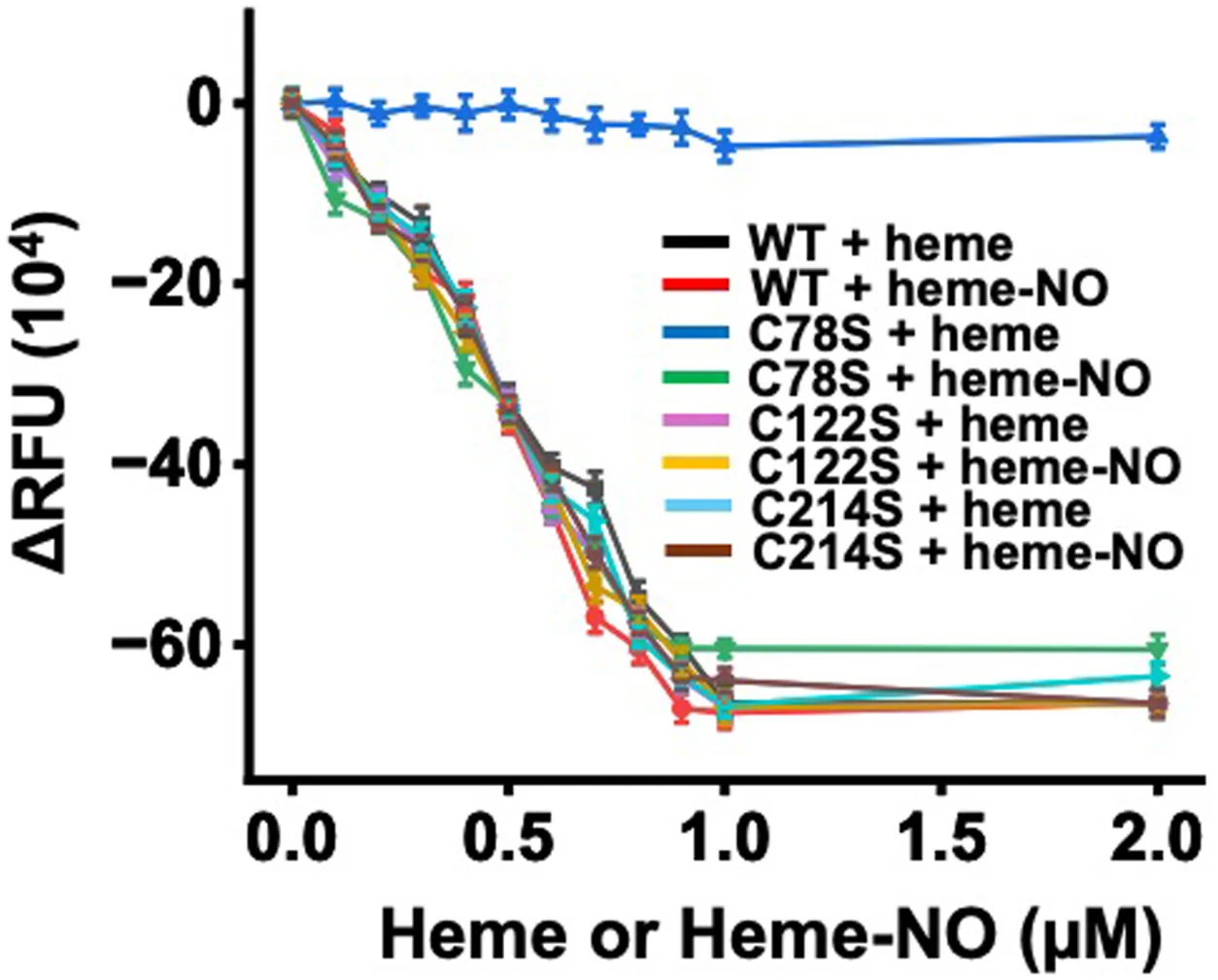
**Binding of ferric heme (heme) or ferrous heme-NO (heme-NO) upon their titration into solutions of the indicated purified and FlAsH-labelled apo-TC-sGCβ_1-385_ proteins.** Fluorescence readings were recorded after the solutions of each purified protein (1 μM final) were incubated with the indicated heme species to give the indicated final concentrations. Heme, ferric heme; heme-NO, ferrous heme NO; RFU, relative fluorescence units. The traces are the mean ± S.D. of three wells and are representative of three independent experiments.

**Figure 6:**
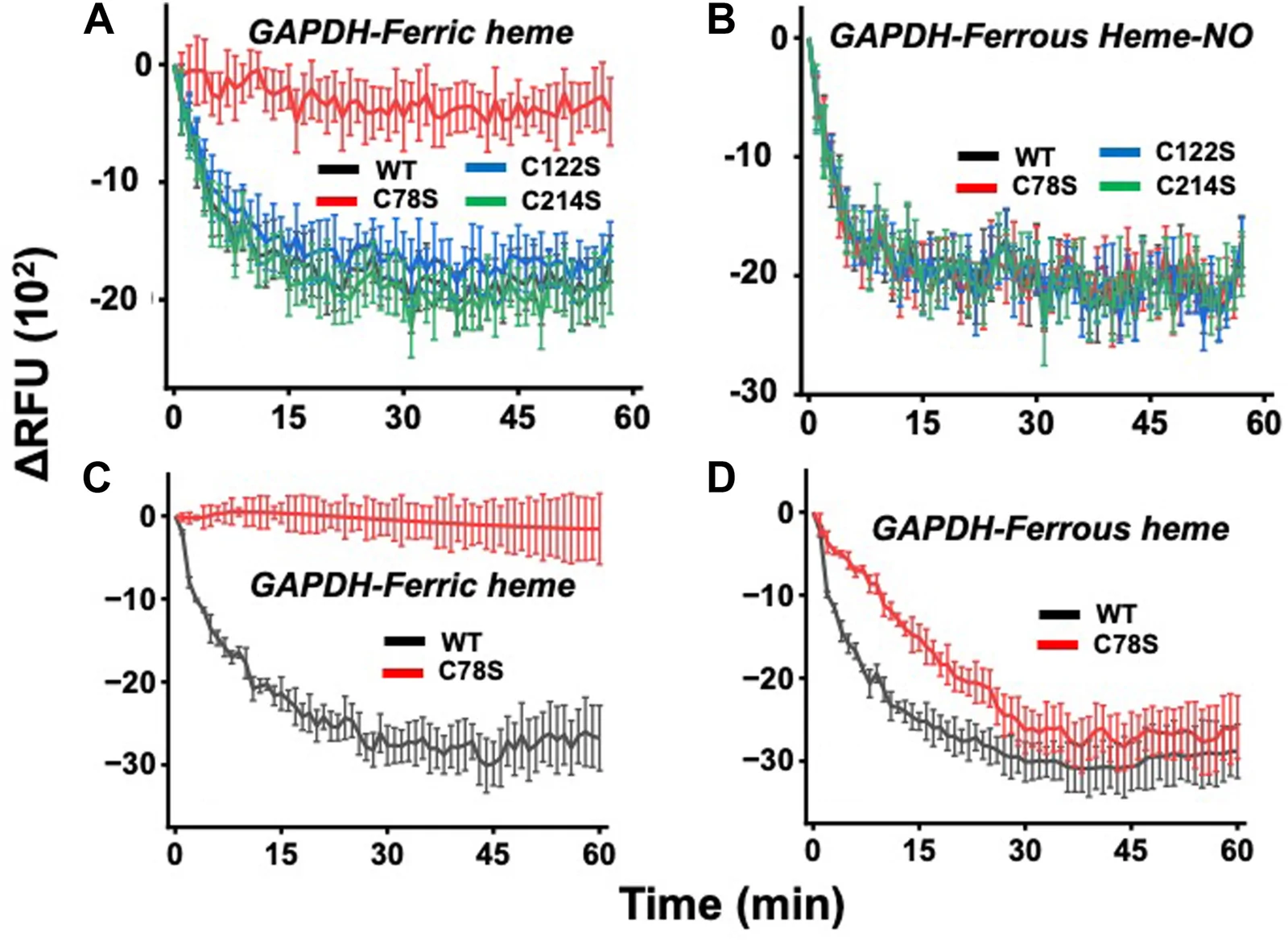
**Kinetics of ferric heme, ferrous heme, or ferrous heme-NO transfer from GAPDH into the apo-TC-sGCβ_1-385_ proteins.** A solution of each indicated FlAsH-labeled apo-TC-sGCβ_1-385_ protein (1 μM final) received either a pre-made ferric heme, ferrous heme, or ferrous heme-NO GAPDH complex (1 μM tetramer final) and the fluorescence intensity was monitored versus time at 25 C. Panels *A*, *B*- experiments were done under air saturated conditions. *C* and *D*, experiments were done under N_2_ atmosphere. RFU, relative fluorescence units. The traces are the mean ± S.D. of three wells and are representative of three independent experiments.

### Oxidation of C78 accompanies ferric heme incorporation into apo-sGCβ

We next studied GAPDH-ferric heme transfer reactions to our sGCβ_1-385_ proteins in the presence of the fluorescent probe 5,5-dimethyl-1,3-cyclohexanedione-fluorescein (DCPFL) which covalently and specifically reacts with and detects oxidized Cys residues as sulfenic acids in proteins (31, 32). We used DCPFL to detect any Cys residues in the apo-sGCβ_1-385_ proteins that might become oxidized (ultimately to their sulfenic acids) during incorporation of ferric heme from GAPDH. Reactions were initiated by mixing either heme-free GAPDH (control) or GAPDH-ferric heme complex with either wild type apo-sGCβ_1-385_ or each of the C78S, C122S, or C214S variants. We also carried out reactions using the Y135A/R139A apo-sGCβ_1-385_ variant, which serves as an additional sGCβ-based control by virtue of it having a non-functional heme-binding site and thus an inability to incorporate heme (13). The reactions were run for 30 min in the presence of DCPFL and analyzed by SDS-PAGE, fluorescence imaging and Coomassie protein staining. Figure 7*A* shows a representative gel image confirming that the reactions all contained similar levels of the individual apo-sGCβ_1-385_ proteins and GAPDH. The representative gel fluorescence image in Figure 7*A* shows that DCPFL bound in the wild-type, C122S, and C214S sGCβ_1-385_ proteins during the GAPDH-ferric heme transfer reactions, indicating that Cys sulfenic acids had formed in them, while DCPFL did not bind in the C78S or Y135A/R139A sGCβ_1-385_ variants. No DCPFL binding was detected in any of these apo-sGCβ_1-385_ proteins when their reactions were run using heme-free GAPDH. Figure 7*B* shows the quantification of the DCPFL binding results for the various reactions. Thus, a Cys oxidation event did or did not occur in the various apo-sGCβ_1-385_ proteins in correlation with their abilities to incorporate the ferric heme provided by GAPDH. These results are consistent with ferric heme uptake into apo-sGCβ_1-385_ specifically depending on concurrent C78 oxidation within the protein.

**Figure 7:**
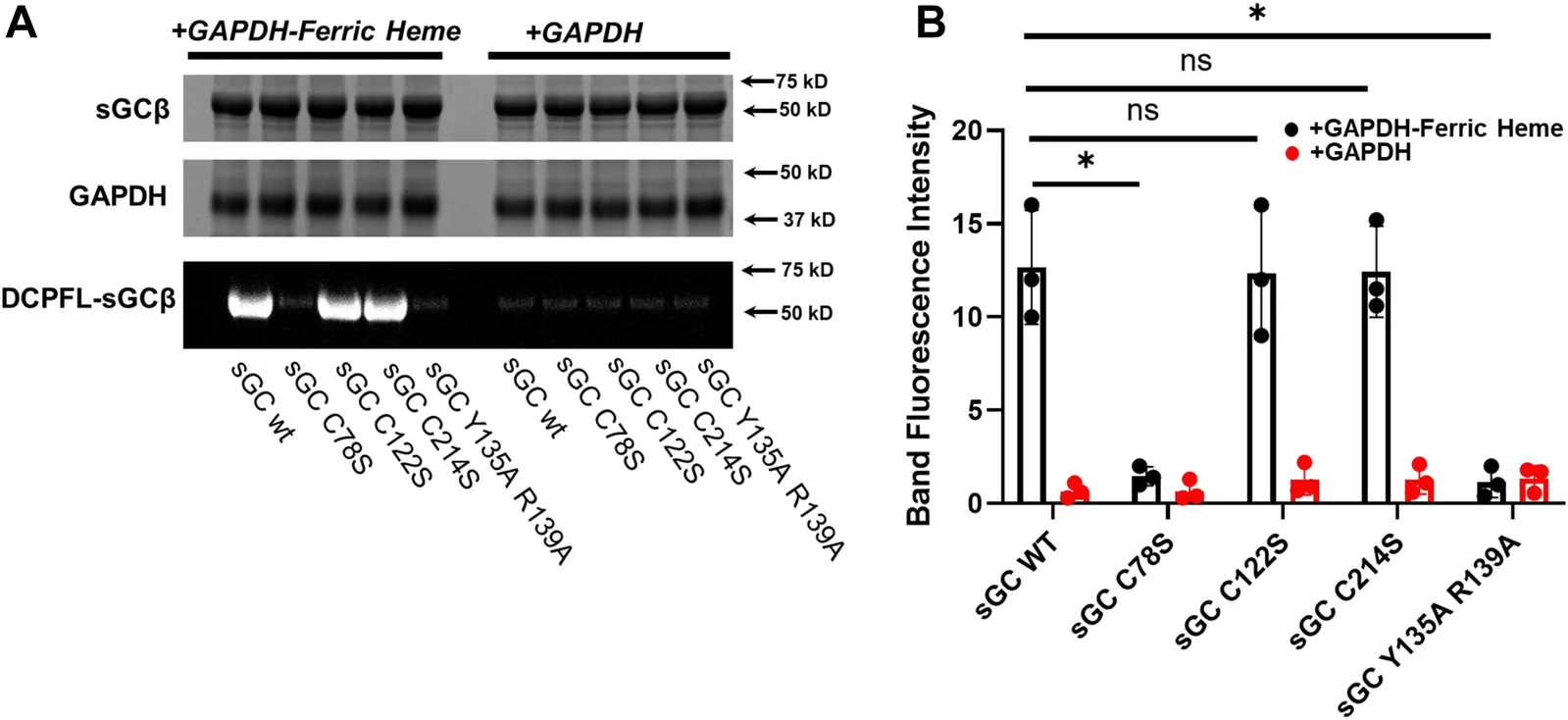
**Oxidation of the C78 thiol in apo-sGCβ_1-385_ occurs during ferric heme transfer from GAPDH.** A solution of each indicated apo-sGCβ_1-385_ protein (1 μM final) and 10 μM DCP-FL was mixed either with GAPDH alone or with a GAPDH ferric heme complex (1 μM tetramer final) and were then incubated for 30 min at room temperature followed by SDS-PAGE analysis. *A*, representative gels comparing the relative levels of the sGCβ_1-385_ and GAPDH proteins present in the reactions as indicated by Coomassie staining (*upper two panels*) and indicating the relative levels of DCP-FL bound in each sGCβ_1-385_ protein after stopping the reactions, as indicated by the white fluorescence intensity (*lower panel*). *B*, quantifications of the fluorescent band intensities for the indicated sGCβ_1-385_ proteins from the indicated reactions. The bars are the mean ± SD of the fluorescence intensities measured in three independent experiments. GraphPad Prism 10 was used to evaluate the differences between groups by a one-way ANOVA. Dunnett's test is used as *post hoc* test. ∗*p* < 0.05 was considered statistically significant in all analyses. ns, not significant.

## Discussion

In a recent study we found that stable heme incorporation into purified apo-sGCβ was associated with, and appeared to require, a one-electron reduction of the incoming ferric heme to its ferrous state that appeared to occur within the sGCβ protein itself (15). This finding implied that redox-labile protein residues in apo-sGCβ may directly participate in and regulate its heme loading. In our current study, we explored whether any of three highly conserved sGCβ Cys residues (C78, 122, 214) participate in this redox-regulated heme incorporation. We determined that only C78 is critical in enabling the incorporation of mitochondrially generated heme or externally supplied ferric heme into apo-sGCβ in live HEK293 cells. In contrast, C78 was not required if a stable form of ferrous heme (ferrous heme-NO) was provided to the cells; in this circumstance, the C78S sGCβ variant incorporated the heme moiety normally and went on to dissociate its Hsp90 and associate with sGCα to form a catalytically functional heterodimer. In our *in vitro* purified protein system that utilizes truncated sGCβ constructs, the C78S variant exhibited a similar dichotomy between its ability to incorporate ferric *versus* ferrous forms of heme. In this context, we also determined that the transfer of GAPDH-bound ferric heme into apo-sGCβ is accompanied by a Cys oxidation event in the apo-sGCβ that is only observed if C78 is present. Our findings in both the purified protein system and in cultured mammalian cells establish that a ferric-to-ferrous heme conversion inside apo-sGCβ is required for it to acquire heme being delivered by GAPDH. Moreover, only C78 undergoes oxidation during the heme incorporation, and is likely the sole source of the reducing equivalent for the ferric to ferrous heme transition inside apo-sGCβ. Thus, under typical mammalian cell culture conditions such as we deployed, we can infer that the heme present inside a mature functional sGC heterodimer becomes ferrous by virtue of a C78-driven reduction within the apo-sGCβ subunit during its heme intake, and does not manifest through: (1) ferrous heme forming before or after it binds to GAPDH in the cells or (2) ferric heme being stably incorporated into a sGCα/β heterodimer followed by heme reduction to ferrous. An illustration showing how the C78-mediated heme reduction fits into the multistep process of sGC maturation in cells is presented in Figure 8.

**Figure 8:**
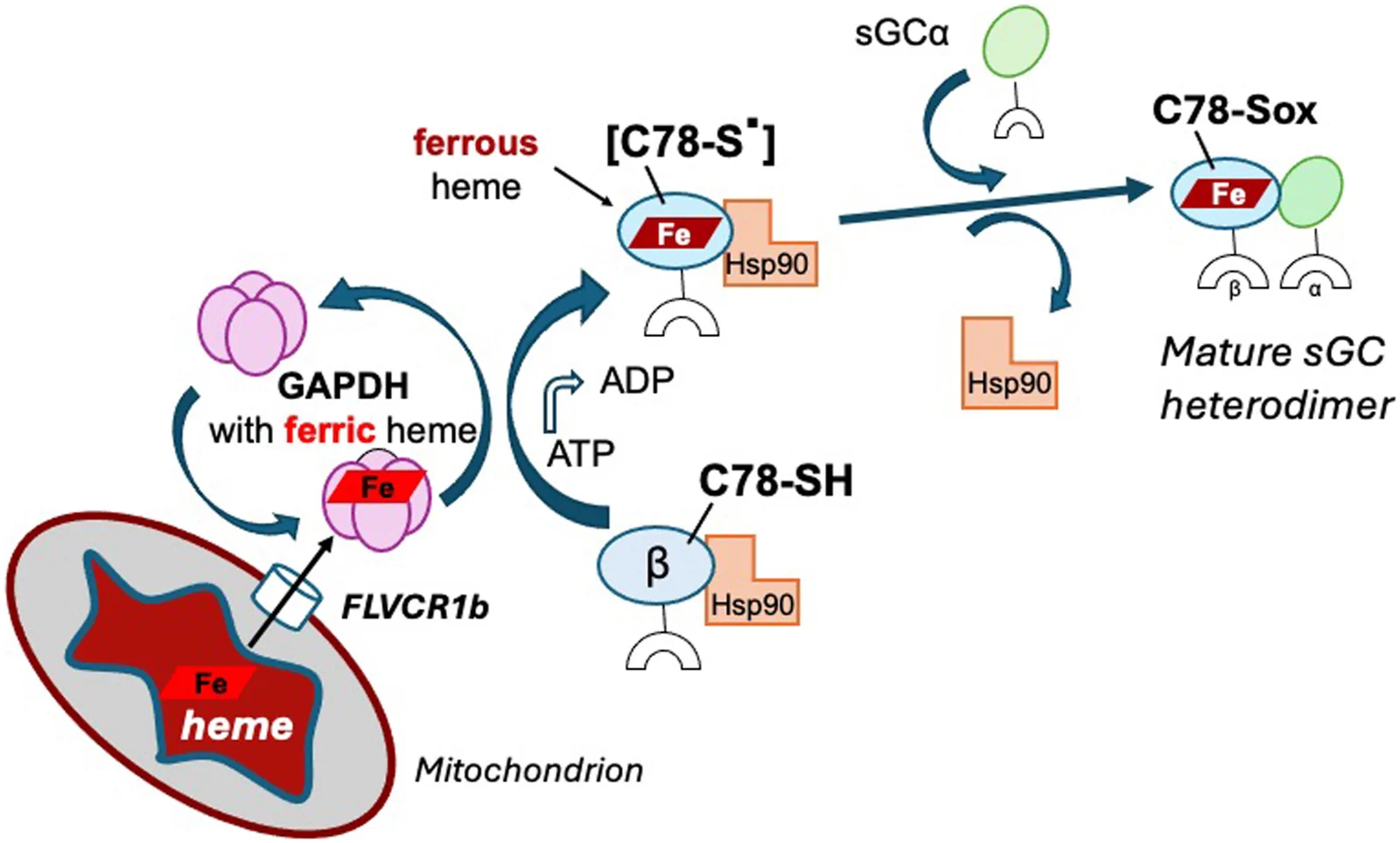
**Redox role of Cys78 during the multi-step sGC maturation process in mammalian cells.** Heme exits the mitochondria through the FLVCR1b exporter and is in ferric form when it transfers into GAPDH. GAPDH the delivers the ferric heme to an apo-sGCβ subunit that is in complex with the Hsp90 chaperone. Stable heme incorporation requires it be reduced from ferric to ferrous inside the sGCβ by the C78 residue, and also requires the ATPase activity of the bound Hsp90. Once the ferrous heme has incorporated into sGCβ, the Hsp90 dissociates and can be replaced by an sGCα subunit to create the mature and functional sGC heterodimer. The fate of the oxidized C78 residue is unknown.

Our experiments with the live HEK293 cells showed that C78 was needed for heme incorporation into apo-sGCβ both when the cells were provided with exogenous ferric heme or when their mitochondrial heme biosynthesis was stimulated. The requirement for C78 under the latter circumstance implies that the mitochondrially generated heme in the HEK293 cells was delivered by GAPDH to apo-sGCβ in its ferric oxidation state. Thus, our findings with C78S apo-sGCβ touch on a long-standing fundamental question in mammalian cell biology; namely, what is the redox state of the mitochondrially generated heme during its transport in cells (21, 33)? Although GAPDH transport of ferric heme appeared to predominate in the HEK293 cells under typical culture conditions, whether this also holds for mitochondrial heme delivery to apo-sGCβ in other cell types or in animal tissues will require further investigation. In any case, because the C78S apo-TC-sGCβ variant can be used to distinguish the redox state of the available (deliverable) heme in cells, it could conceivably be used to interrogate the redox state of the GAPDH-heme pool in any cell type or tissue under different biological conditions.

Mechanistically, it is perhaps not surprising that among the three Cys residues we studied, the one that is positioned closest to the sGCβ heme (C78, Fig. 1) provides the reducing equivalent to the incoming ferric heme. Indeed, based on the Moser-Dutton ruler (34, 35), C78 is positioned well within the distance range that would allow a direct electron transfer to the heme ring. As noted, none of the three Cys we studied are positions to form disulfides. Together, this suggests a simple mechanism whereby the incoming ferric heme transiently occupies the sGCβ heme pocket and this triggers a one-electron transfer from the C78, forming its thiol-centered radical in the process, which then goes on to become further oxidized to its sulfenic acid. Once the heme iron has been reduced to ferrous its binding within the sGCβ heme pocket becomes significantly stabilized. A one-electron reduction of the ferric heme by C78 is consistent with the strong thermodynamic gradient that exists between the oxidation of protein Cys residues, whose reduction potentials range from −325 to −178 mV (36, 37) and reduction of the sGCβ ferric heme, which has a positive reduction potential (+187 mV) (38). There also are precedents for thiol-mediated heme reduction occurring in proteins (39, 40, 41). Indeed, a previous study of purified sGC heterodimer showed that, within its sGCβ subunit, C78 and C122 oxidation could occur during the process of reductive nitrosylation of the bound ferric heme in response to excess NO (42). In that study, the ferrous heme in a purified sGC heterodimer was first oxidized to ferric by ODQ addition and then received excess NO, which resulted in the sGC containing ferrous heme-NO and S-nitrosated C78 and C122 residues. Mechanistically, this implied that both C78 or C122 could perform a one-electron reduction of the initially formed ferric heme-NO species, which generated the ferrous heme-nitrosyl species and the Cys radical, which then quickly reacted with the excess NO to become S-nitrosated (42). Similar Cys-driven heme reductions occur in hemoglobin, in which a Cys positioned near the heme can reduce the ferrous heme-O_2_ species to generate met-Hb and superoxide (43) and, in the presence of NO, can form its Cys nitrosothiol species (44). In studies in which Cys residues were intentionally incorporated into myoglobin at various distances from its heme, the oxidation rates of the individual Cys inversely correlated to their distances from the bound heme after CO was added to prompt Cys reduction of the ferric heme iron to form the ferrous heme-CO species (45, 46). But there are two important facets that distinguish our current findings with apo-sGCβ from these other studies: (1) Specificity—In our study no other Cys residue in sGCβ besides C78 enabled the incorporation of ferric heme and the accompanying Cys oxidation event. Of note, the C78 specificity held in live cells for heme incorporation into apo-sGCβ, despite cells containing many Cys-containing proteins and abundant low molecular weight thiols like glutathione. (2) Driving force—In the examples noted above for myoglobin, sGC, and hemoglobin, internal Cys residue oxidations did not occur spontaneously but instead depended on the Cys oxidation event being coupled to the ability of the protein’s heme to bind a preferred ferrous heme ligand (NO, CO) or to the release of a bound ligand (release bound O_2_ as superoxide). In fact, the C78-driven heme reduction in sGCβ that is obligatory for its stable incorporation both in live cells and *in vitro* took place in the absence of added heme ligands like NO or CO, implying that it is driven simply by the apo-sGCβ′s strong binding preference and higher affinity toward ferrous over ferric heme (47). To our knowledge, relying on a Cys-driven, internal one-electron reduction for heme incorporation makes sGCβ unusual regarding its mechanism of heme incorporation. Indeed, while a heme redox step is also known to enable its transfer into some bacterial cytochromes, in that case the heme must undergo the opposite redox transformation (ferrous to ferric) to become incorporated (48).

The prominent role of C78 in driving a redox step that is critical for heme uptake and consequent sGC maturation raises fascinating follow-up questions: After C78 becomes oxidized, does this modulate sGC catalytic function? Interestingly, our current work suggest it may not, given that the sGCβ variant that is entirely missing C78 could still form a catalytically proficient sGC heterodimer in cells when a ferrous form of heme was provided. Is the oxidized C78 ever reduced back to its thiol form in the heterodimer, and if so, would the mechanism involve a cytochrome b5 reductase enzyme, which is known to mediate sGC ferric heme reduction in the heterodimer (49)? This would allow C78 to possibly perform a “redox rescue” when the ferrous heme in sGC becomes oxidized to ferric by cellular oxidative stress. These and other questions can now be explored. In a broader context, we know that mammalian cells and tissues naturally maintain a heme deficit (25, 50) that leads to a significant percentage of their hemeproteins like sGCβ existing in their apo forms (5, 25, 51). Under this circumstance, cell heme allocation into the apo-sGCβ sub-population is stimulated by biological NO exposure (16, 25). It will now be interesting to examine whether C78 is also required in this circumstance. In any case, uncovering the internal C78 redox reaction in sGC and its importance helps advance our understanding of how sGC matures in cells to its functional form and also suggests a plausible way that its heme acquisition could link to the cell’s overall thiol redox status, which is well known to impact sGC activity (22, 52, 53). This can now be addressed.

## Experimental procedures

### General methods and materials

The TC-FlAsH Tetra cysteine Tag Detection Kit was obtained from Invitrogen. DCP-FL was a gift from Xoder Technologies, Inc, Winston-Salem, NC. BAY 41-2272 (5-cyclopropyl-2-[1-(2-fluorobenzyl)-1H-pyrazolo[4-b]pyridin-3-yl]pyrimidin-4-ylamine, or BAY41) and BAY, 58-2667 (4-[[(4-Carboxybutyl)[2-[2-[[4-(2-phenylethyl)phenyl]methoxy]phenyl]ethyl] amino]methyl]benzoic acid hydrochloride, Cinaciguat, or BAY58) were obtained from Sigma. The ferrous heme-NO reagent was generated and provided by Dr Nicolai Lehnert, U. Michigan, and details of its generation and characterization were reported previously (28). Briefly, in a N_2_ filled glovebox, [Fe(protoporphyrin IX)Cl] and sodium methoxide were dissolved in dry, N_2_ purged methanol. The complex was then reduced to the corresponding ferrous complex by the addition of ethanethiol. In a Schlenk flask, the solution was then exposed to NO gas and allowed to stir for several minutes resulting in a noticeable change to a darker, red colored solution. The solution was then transferred back into the glovebox and precipitated with diethyl ether and storing in a −20 °C freezer overnight. The resulting solid was collected and washed with methanol followed by diethyl ether before allowing it to dry in the glovebox. The resulting dry solid was collected and stored in the glovebox or a −80 °C freezer until use. All other reagents and materials were obtained from sources reported elsewhere (13, 15).

### Molecular biology

pET20b expression plasmid containing rat sGCβ_1–385_ with a C-terminal His6 tag was a gift from Dr Michael Marletta (University of California) (15, 54). pET20b expression plasmids containing rat sGCβ_1–385_ with a TC motif (CCPGCC) inserted at residue 239 to 244 (TC-sGCβ_1–385_) (15), sGCβ_1–385_ Y135A R139A (15), human GAPDH containing a TC motif to bind FlAsH (TC-GAPDH) (15), and human heat shock protein 90β (Hsp90) (55) were reported previously. pET20b expression plasmids containing rat sGCβ_1–385_ or TC-sGCβ_1–385_ with C78S, C122S, or C214S single point mutation were made with primers listed in Table S1 and Q5 High-Fidelity DNA Polymerase using rat sGCβ_1–385_ and TC-sGCβ_1–385_ as templates. pCMV5 expression plasmid contain rat TC-sGCβ_1–619_ was reported previously (13). pCMV5 expression plasmid containing rat TC-sGCβ_1–619_ with C78S, C122S and C214S single-point mutation were made with primers listed in Table S1 and Q5 High-Fidelity DNA Polymerase using pCMV5 rat sGCβ_1–619_ as templates. pCMV5 plasmid containing rat sGCα_1-690_ was reported previously (13). pGST-parallel1 vector Human GAPDH containing a TC motif is reported previously (56).

### Protein purification

His6-tagged rat sGCβ_1–385_ and GST-tagged human GAPDH were expressed in *E. coli* BL21(DE3) and purified using methods reported previously (29).

### Cell culture and transient transfection

HEK293 cells (Fisher, Cat# 033377X) were cultured on fluorescent 96-well plates or on 10-cm dishes as described elsewhere (20). Plasmids coding for full-length sGCα(aa 1-690), TC-sGCβ proteins and TC-GAPDH were transfected into HEK293 cells using a method reported previously (20). Briefly, WT and mutant sGC1(1–385) constructsβ were each transformed into *E. Coli* BL21 cells for expression. Overnight cultures were grown at 37 °C in Luria broth and used to inoculate 500-ml expression cultures grown in Terrific broth. Expression cultures were grown until A600 reaches 0.5 and subsequently cold-shocked for 10 min in an ice bath. Protein expression was induced with 250 μM isopropyl-D-1-thiogalactopyranoside for 20 h at 25 °C at 250 rpm. Cells were harvested by centrifugation and then resuspended in 40 mM EPPS, 150 mM NaCl, 10% glycerol, pH 7.5, buffer supplemented with protease inhibitors, benzonase, and lysozyme. Resuspended cells were lysed by sonication and clarified by centrifugation. Lysate was then purified with a gravity nickel-nitrilotriacetic acid column. GAPDH were expressed using GST constructs in *E. coli.* and grown initially in100 ml luria broth for 16 h at 37 °C, followed by inoculation in 2 L of terrific broth. Cells were induced with 1 mM IPTG after the cell density reached to 0.8 to 1.0 OD and were incubated for another 48 h at room temperature. Cells were harvested and purified using glutathione Sepharose according to the manufacturer's instructions (Amersham Biosciences). After thrombin cleavage (37 °C, 1 h), thrombin was removed with HiTrap Benzamidine FF affinity column (GE Healthcare).

### IP and Western blot analysis

IP of sGCβ and Hsp90 was performed using a method reported previously (16). Briefly, anti-sGCβ antibody was used to IP its bound proteins in HEK293 cell supernatant using protein G beads. GAPDH and Hsp90 were then probed using the respective antibodies. PAGE and Western blot analysis were performed using standard procedures. Band intensities were quantified using ImageJ (National Institutes of Health). Relative band intensities are normalized to actin. Primary antibodies and their dilutions used for Western blotting in this study were rabbit polyclonal sGCβ1 antibody, 1:1000 dilution (Abcam Cat# ab84955); rabbit polyclonal sGCα antibody, 1:1000 dilution (Cayman Chemical Cat# 160895,); rabbit polyclonal human Hsp90 antibody, 1:500 dilution (Cell Signaling Technology Cat# 4874); mouse monoclonal β-actin antibody, 1:1000 dilution (Sigma-Aldrich Cat# A5441). Goat anti-mouse IgG (H + L)-HRP conjugate 1:1000 (Bio-Rad Cat# 170-6516). Goat anti-rabbit IgG, HRP conjugate 1:2000 (Cell Signaling Technology, #7074).

### FIAsH labeling of TC-sGCβ and TC-GAPDH

TC-sGCβ expressed in HEK293 cells was labeled with the TC-FlAsH In-Cell Tetra Cysteine Tag Detection Kit (Invitrogen) using a method reported previously (16). Briefly, transfected cells grown in fluorescence 96-well plates were washed three times with phenol red–free DMEM containing 1 g/L glucose. Cells were then incubated with a mixture of FlAsH/1,2-ethanedithiol made in Opti-MEM (final concentration 0.5 μM/12.5 μM) for 1 h at room temperature. Afterward, cells were washed three times with 250 μM 1,2-ethanedithiol in phenol red-free DMEM with 10% FBS and then three times with phenol red–free DMEM with 10% FBS. Purified TC-sGCβ_1-385_ and mutant proteins were labeled with the TC-FlAsH In-Cell Tetra Cysteine Tag Detection Kit using a method published elsewhere (15). Briefly, TC-sGCβ_1-385_ proteins were reduced with 1 mM TCEP and for 30 min under RT then incubate with 1.1 equivalents of FlAsH/1,2-ethanedithiol in PBS pH 7.4 for 1 h at RT in dark. The reaction mixture were then applied to PD10 column to remove the excessive reagents.

### Monitoring heme transfer into TC-sGC in cells

Heme insertion into FlAsH-labeled TC-sGCβ (1–619) or the FlAsH-labeled TC-GAPDH proteins expressed in HEK293 cells was monitored at 37 °C on a Flexstation3 96-well plate reader instrument as reported previously (16). Briefly, the FlAsH-TC proteins were excited at 488 nm, and emission was detected at 528 nm. 100 readings were taken for signal integration every 1 min. Ferric heme or ferrous heme-NO additions (5 μM), or δ-ALA (500 μM) and Fe-cit (200 μM) additions, were done to initiate the heme delivery into the FlAsH-labeled proteins in the cells.

### Titration of cell supernatants with ferric heme or ferrous heme-NO

Cell supernatants from HEK293 cells expressing FlAsH-labeled apo-TC-sGCβ proteins were prepared and supernatant aliquots (0.5 mg total protein) were incubated with increasing concentrations of ferric heme or ferrous heme-NO in 96-well plates. Mixtures were incubated under RT for 30 min, the plates were excited at 488 nm, and the emission intensities were detected at 528 nm and used to obtain titration curves.

### Monitoring heme transfer into purified TC-sGCβ_1-385_ proteins and anerobic heme transfer experiments

Ferric heme transfer from GAPDH heme complex into purified FlAsH-TC-apo-sGCβ_1-385_ proteins was monitored by methods described previously (15). Briefly, reactions were run at room temperature in black masked 96-well fluorescence plates. Wells contained PBS, pH7.4 and 1 μM of FlAsH-TC-apo- sGCβ_1-385_ protein. GAPDH–heme complex was added to start the reaction (final concentration 1 μM), and the fluorescence emission was monitored over time in an iD5 max fluorescence microplate reader (Molecular Devices) with excitation/emission set at 488 nm and 530 nm. Ferrous heme transfer from GAPDH heme complex into FlAsH-TC-apo-sGCβ_1-385_ proteins was monitored in anaerobic fluorescence cuvettes using method described previously (15). Briefly, solutions of FlAsH-TC-apo-sGCβ_1-385_ protein and GAPDH ferric heme complex separately underwent repeated vacuum and N_2_ cycling using a gas train and put under N_2_ atmosphere. The GAPDH ferric heme complex was reduced to ferrous by anaerobic dithionite addition and was transferred anaerobically into an anaerobic fluorescence cuvette containing 1 μM FlAsH-TC-apo-sGCβ_1-385_ under N_2_. The fluorescence emission was monitored *versus* time using a SF-2500 Fluorescence Spectrophotometer (Hitachi) with excitation/emission set at 490 nm and 530 nm.

### Detecting protein Cys-sulfenic acid residues with fluorescence probes

Oxidized Cys residues generated as a consequence of heme transfer from GAPDH to apo-sGCβ_1-385_ proteins was detected by a published method with modifications (29). Briefly, the purified apo-sGCβ_1-385_ proteins were first incubated with 1 mM TCEP to fully reduce all Cys thiols. Excess TCEP was removed by passing thought a desalting PD-10 column. The fluorescence probe DCP-FL (10 μM final) (31) was added along with GAPDH-ferric heme complex (1 μM tetramer final) to each apo-sGCβ_1-385_ protein (1 μM final), and the reaction mixture was incubated for 30 min at RT. The products were then run on SDS-PAGE. In-gel fluorescence was imaged using a Chemdoc instrument (Bio-Rad).

### cGMP ELISA

The cGMP concentration in cell supernatant assays was determined by a method described previously (57). Briefly, GTP (0.25 mM), MgCl_2_ (0.5 mM), phosphodiesterase inhibitor 3-isobutyl-1-methylxanthine (0.5 mM), sGC stimulator BAY 41 (10 μM) or sGC activator BAY 58 (10 μM) were mixed with cell supernatant samples and incubated for 30 min at 37 °C. The cGMP produced was quantified using a cGMP ELISA kit (Cell Signaling Technology). Absorbance values at 450 nm were read on a Flexstation3 96-well plate reader (Molecular Devices). cGMP production values were normalized according to the total protein amounts in the cell supernatants.

### Statistics and data analysis

Data analysis was performed using Origin Lab 2022 or GraphPad Prism 10 (Dotmatics). Experiments were done in two or three independent trials as indicated in the figure legends. GraphPad Prism 10 was used to evaluate the differences between groups by a one-way ANOVA. Dunnett's test is used as *post hoc* test. *p* < 0.05 was considered statistically significant in all analyses.

### Molecular modeling

Structures of sGC heterodimers (9, 10) were visualized, rendered, and used for intraprotein distance measurements using open-source PyMol v. 3.1.0 (Schrödinger, LLC. The PyMOL Molecular Graphics System, Version 3.10). Cys surface accessibility was calculated using GETAREA (58), with <20% accessible area considered buried while those with 20 to 50% accessible area and >50% accessible area are respectively labeled as moderately and highly accessible.

## Data availability

All data are either contained within the article or are available from the corresponding Author (Dennis Stuehr, stuehrd@ccf.org) upon reasonable request.

## Supporting information

This article contains supporting information.

## Conflict of interest

The authors declare that they have no conflicts of interest with the contents of this article. The content is solely the responsibility of the authors and does not necessarily represent the official views of the National Institutes of Health.
