# Supplementary material for: Soluble guanylyl cyclase maturation in cells depends on reduction of the incoming heme by a cysteine in the enzyme’s beta subunit
Source: J Biol Chem. 2026 Sep 8;302(11):113541. doi: 10.1016/j.jbc.2026.113541 (PMC13681641; doi:10.1016/j.jbc.2026.113541)
Supplement: Supplementary file 1 [file mmc1.pdf]

## Table. S1. Primers used to make sGC mutants

| Mutants | Primers |
|---|---|
| pCMV5 rat TC-sGC$\beta_{1-619}$ C78S<br>pET20b rat sGC$\beta_{1-385}$ C78S<br>pET20b rat TC-sGC$\beta_{1-385}$ C78S | 5'-gtatcatagccagactcttggcagaagacgaaaaacatctt-3'<br>5'-aagatgtttttcgtcttctgccaagagtctggctatgatac-3' |
| pCMV5 rat TC-sGC$\beta_{1-619}$ C122S<br>pET20b rat sGC$\beta_{1-385}$ C122S<br>pET20b rat TC-sGC$\beta_{1-385}$ C122S | 5'-ctttttctgcatcggtacaccggaaggaaggtg-3'<br>5'-caccttccttccggtgtaccgatgcagaaaaag-3' |
| pCMV5 rat TC-sGC$\beta_{1-619}$ C214S<br>pET20b rat sGC$\beta_{1-385}$ C214S<br>pET20b rat TC-sGC$\beta_{1-385}$ C214S | 5'-tgaaaaggaaacgctttacagaaggtgtacgggct-3'<br>5'-agcccgtacaccttctgtaaagcgtttccttttca-3' |

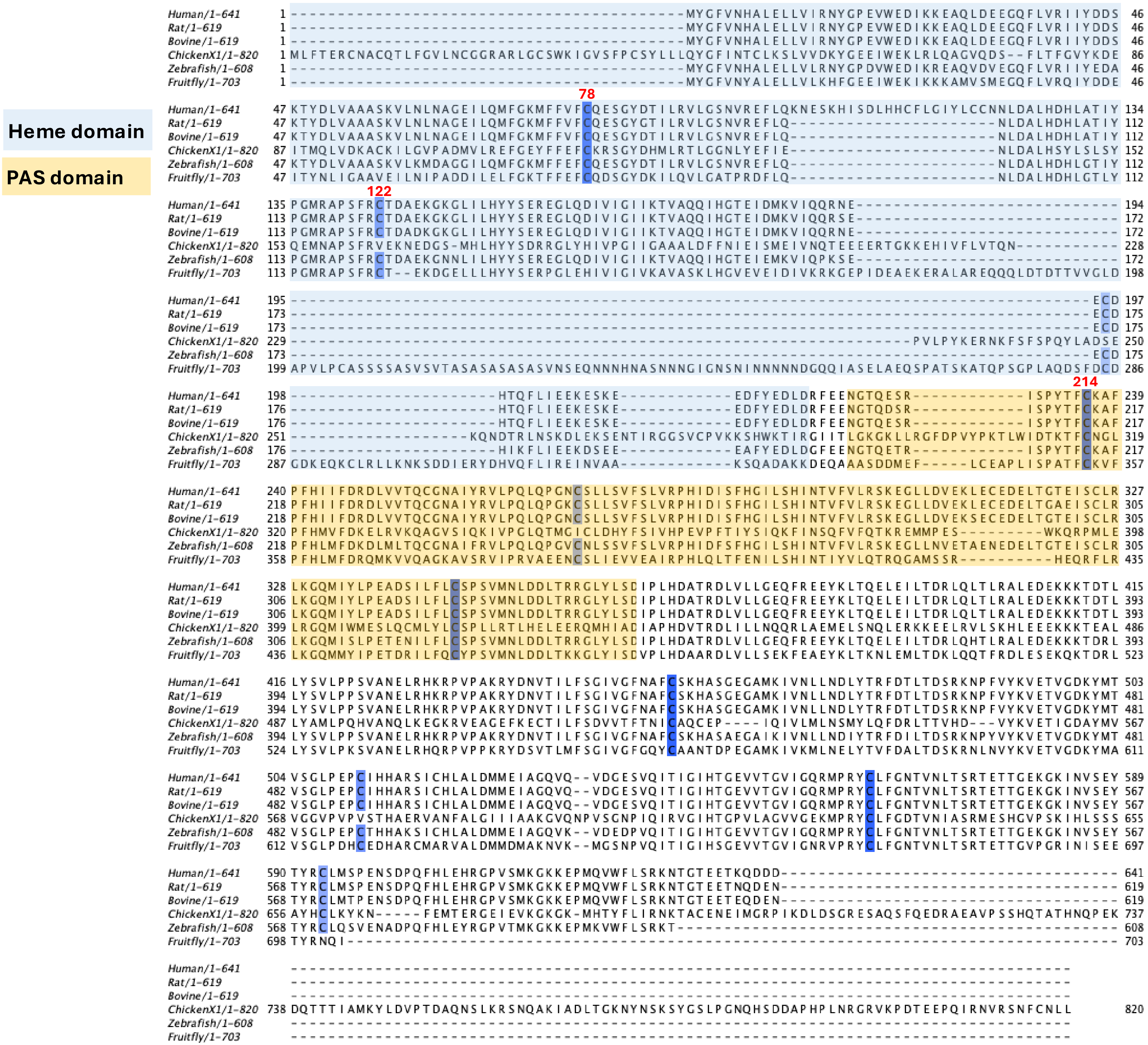


**Fig. S1. Multiple sequence alignment of sGCβ proteins from the indicated species.** The sequences were downloaded from NCBI (Human, NP_001278880.1; Rat , BAC55087.2; Bovine, NP_777066.1; Chicken, XP_052542358.1; Zebrafish, NP_001238874.1; Fruitfly , XP_062125798.1) and aligned in Jalview (University of Dundee, Dundee, Scotland) using Clustal Omega (European Bioinformatics Institute, Cambridgeshire, UK). Cys residues are highlighted in purple with shade intensity is proportional to their degree of conservation. Cys residues studied in our report are noted in red, with numbering according to rat sGCβ. Heme domain is shaded with light blue and PAS domain is shaded with light yellow.

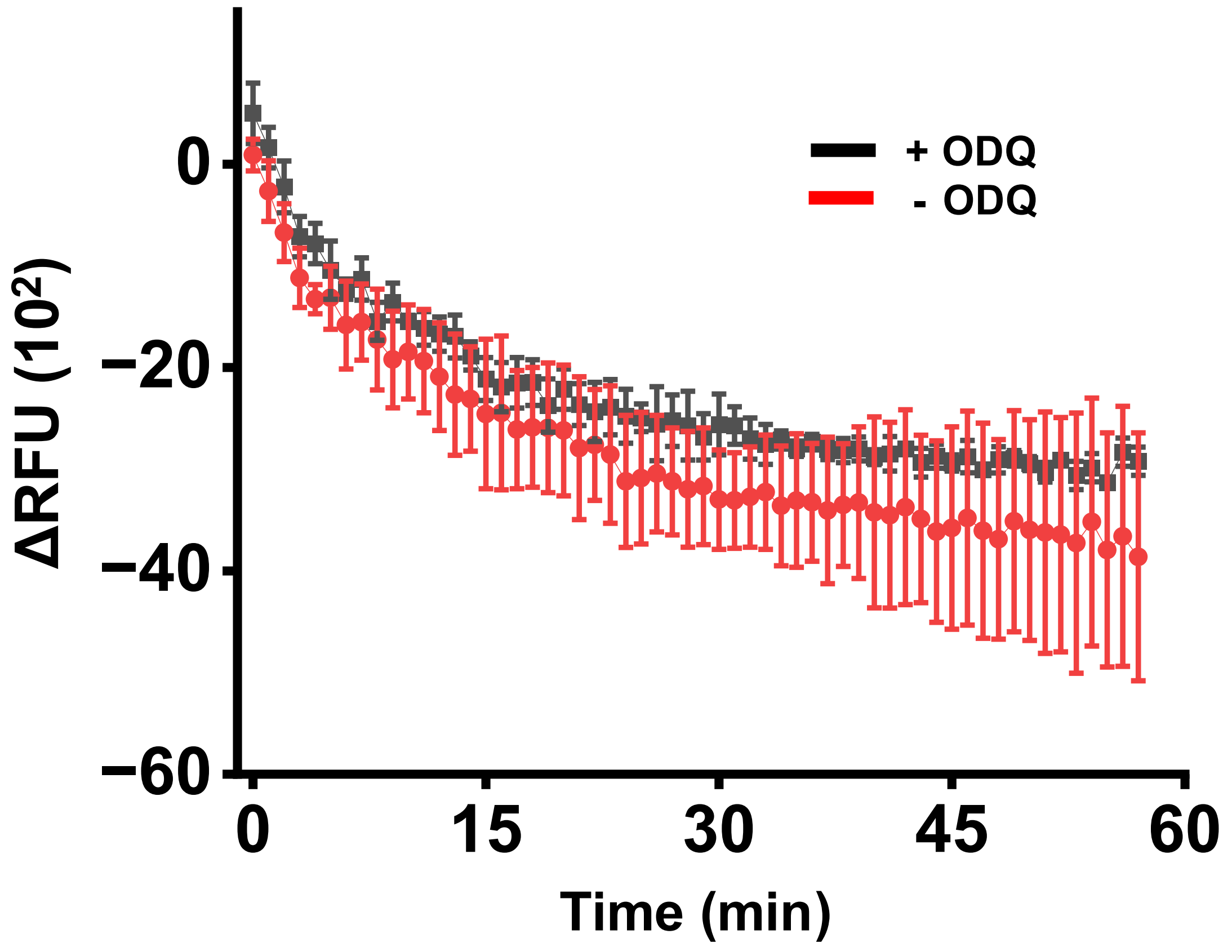


**Fig. S2. ODQ effect on heme loading into GAPDH in cells upon triggering mitochondrial heme production.** HEK293 cells transfected to express TC-GAPDH were FlAsH labeled and then received δALA/Fe to stimulate their heme biosynthesis in the absence or presence of ODQ. Fluorescence was then recorded vs time, and a drop in fluorescence intensity indicates an increase in TC-GAPDH heme content. Data are the mean +/- SD for 3 replica wells.

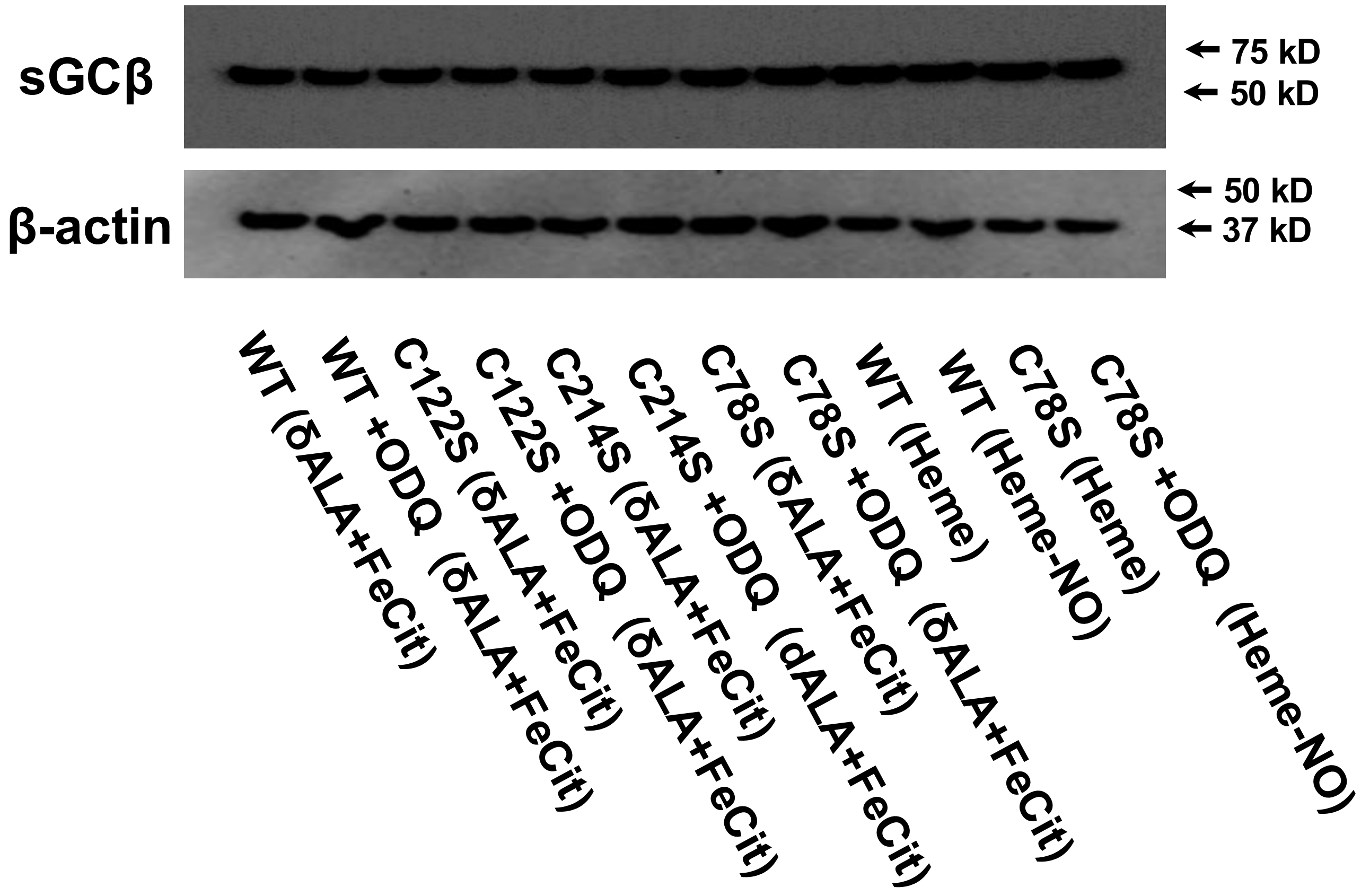


**Fig. S3. Relative expression levels of the indicated sGCβ proteins in cells under the indicated experimental conditions from Fig. 2.** Relative sGCβ protein expression levels in samples of cell supernatants containing equal total protein were determined by SDS-PAGE and Western blot. Western shown is representative of three independent experiments.
